# Early interventional treatment with intranasal corticosteroids is superior to post-onset treatment in Japanese cedar/cypress pollinosis

**DOI:** 10.1186/2045-7022-3-S2-P36

**Published:** 2013-07-16

**Authors:** Takenori Haruna, Mitsurhiro Okano, Takaya Higaki, Yasuyuki Noyama, Misato Hirai, Kazunori Nishizaki

**Affiliations:** 1 Okayama University Graduate School of Medicine, Dentistry and Pharmaceutical Sciences, Okayama, Japan; 2 Okayama University Graduate School of Medicine, Dentistry and Pharmaceutical Sciences, Otolaryngology-Head & Neck Surgery, Okayama, Japan; 3 Okayama Saiseikai General Hospital, Otolaryngology-Head & Neck Surgery, Okayama, Japan

## Background

The usefulness of early interventional treatment (EIT) with intranasal corticosteroids (INS) as compared to post-onset treatment (POT) has not been clarified. We sought to determine the efficacy and safety of EIT with INS compared with POT and placebo in Japanese cedar/cypress pollinosis.

## Method

We designed a three-armed, double-blinded, randomized, placebo-controlled trial. Patients received mometasone furoate nasal spray (EIT group: n=25), placebo (n=25), or 4 weeks of placebo followed by 8 weeks of mometasone (POT group: n=25) for a 12-week period starting on February 1, 2011. The primary endpoint was the comparison of the total nasal symptom score (TNSS) among the three groups. Total ocular symptom score (TOSS), total naso-ocular symptom score (TSS), ARIA classification, safety, etc. were secondary endpoints.

## Results

The placebo and POT groups, but not the EIT group, showed a significant exacerbation of TNSS and TOSS soon after the start of pollen counts being high on consecutive days. The 12-week average TSS in the EIT group (score, 2.3) was significantly lower than in the placebo (5.0; P<0.01) and POT (3.9; P=0.03) groups. All subjects in the placebo and POT groups were classified as having persistent rhinitis, while 80% of the EIT group met the ARIA classification criteria (P=0.03). QOL score and nasal ECP levels were lower in the EIT and POT groups as compared with the placebo group. Daytime sleepiness, smell disturbance and the average dose of loratadine taken as the rescue medication were similar. Treatment with mometasone was well tolerated.

## Conclusion

EIT with INS is superior to POT in controlling pollinosis.

